# Neurotoxic lesions of ventrolateral prefrontal cortex impair object-in-place scene memory

**DOI:** 10.1111/j.1460-9568.2007.05468.x

**Published:** 2007-04-01

**Authors:** Charles R E Wilson, David Gaffan, Anna S Mitchell, Mark G Baxter

**Affiliations:** Department of Experimental Psychology, Oxford University South Parks Road, Oxford OX1 3UD, United Kingdom

**Keywords:** episodic, macaque, memory, monkey, prefrontal

## Abstract

Disconnection of the frontal lobe from the inferotemporal cortex produces deficits in a number of cognitive tasks that require the application of memory-dependent rules to visual stimuli. The specific regions of frontal cortex that interact with the temporal lobe in performance of these tasks remain undefined. One capacity that is impaired by frontal–temporal disconnection is rapid learning of new object-in-place scene problems, in which visual discriminations between two small typographic characters are learned in the context of different visually complex scenes. In the present study, we examined whether neurotoxic lesions of ventrolateral prefrontal cortex in one hemisphere, combined with ablation of inferior temporal cortex in the contralateral hemisphere, would impair learning of new object-in-place scene problems. Male macaque monkeys learned 10 or 20 new object-in-place problems in each daily test session. Unilateral neurotoxic lesions of ventrolateral prefrontal cortex produced by multiple injections of a mixture of ibotenate and *N*-methyl-d-aspartate did not affect performance. However, when disconnection from inferotemporal cortex was completed by ablating this region contralateral to the neurotoxic prefrontal lesion, new learning was substantially impaired. Sham disconnection (injecting saline instead of neurotoxin contralateral to the inferotemporal lesion) did not affect performance. These findings support two conclusions: first, that the ventrolateral prefrontal cortex is a critical area within the frontal lobe for scene memory; and second, the effects of ablations of prefrontal cortex can be confidently attributed to the loss of cell bodies within the prefrontal cortex rather than to interruption of fibres of passage through the lesioned area.

## Introduction

Interaction between the frontal cortex and inferotemporal cortex is vital for a broad array of cognitive abilities. Conditional visual learning tasks, recognition memory, strategy implementation, discrimination learning set and object-in-place scene memory are all impaired following disconnection of the frontal lobe from the inferotemporal cortex by crossed unilateral lesions (Parker & Gaffan, 1998; Bussey *et al*., 2002; Browning *et al*., 2005, 2006). These impairments occur in the context of preserved associative learning ability, however, because concurrent object–reward discrimination learning is unimpaired after such lesions (Parker & Gaffan, 1998; Gaffan *et al*., 2002). Such impairments can be characterized in a variety of ways, including as disruptions in the ability to rapidly learn new episodic-like memory problems as well as in the ability to apply memory-dependent performance rules to visual objects or to recall these rules but, importantly, they cannot be characterized as a generalized impairment in learning about objects.

An outstanding question concerns whether these impairments are related to the integrative action of the frontal cortex, and in particular the prefrontal cortex, on visual information, or whether particular regions of prefrontal cortex interact in specific ways with inferotemporal cortex to subserve particular functions. Lesions that produce pure double dissociations of function within the prefrontal cortex are extremely rare (Gaffan, 2002), although lesions to subregions of the prefrontal cortex can have devastating effects on particular aspects of cognition. For example, the impairment of working memory following lesions limited to the cortex in the banks of the sulcus principalis (area 46) is well known (e.g. Goldman & Rosvold, 1970).

In this study we were concerned with one aspect of localization of function within prefrontal cortex in respect of its interaction with inferotemporal cortex, as well as with the methodology used to produce subregional lesions within the frontal lobe. The ventrolateral prefrontal cortex, including areas 45 and 47/12 (Petrides & Pandya, 2002), receives extensive visual inputs from the inferotemporal cortex (e.g. Pandya & Yeterian, 1996). Because rapid learning of new object-in-place scene problems, a test of episodic-like memory (Gaffan, 1994), depends on frontal–inferotemporal interaction (Browning *et al*., 2005), and the ventrolateral prefrontal cortex is a major target of inferotemporal efferents, this region is a natural candidate for a critical locus within the frontal cortex for specialization for episodic-like memory. Thus, we determined whether the disconnection of relatively small regions of the ventrolateral prefrontal cortex from inferotemporal input could produce a substantial impairment in object-in-place scene memory.

Because aspiration of cortical tissue may interrupt fibres of passage moving through the cortical region en route to their destination, the prefrontal lesions were produced via injections of excitotoxin rather than by subpial aspiration of grey matter. The aspiration technique has the advantage of reliability, but questions of interpretation after small lesions within the prefrontal cortex could be raised. For example, aspiration of cortical tissue could damage axons of monoaminergic projections coursing through the grey matter of the frontal lobe (Morrison *et al*., 1982). Thus, impairments observed after a small prefrontal lesion could possibly result from a combination of loss of cortical tissue and deafferentation of other regions within the frontal cortex. Because it spares fibres of passage, the neurotoxic lesion technique does not have this difficulty. Thus, a secondary aim of the current experiment was to develop a technique for producing neurotoxic lesions within the prefrontal cortex of the macaque monkey.

## Materials and methods

### Subjects

Two rhesus monkeys (*Macaca mulatta*; S1 and S2) and two cynomolgus monkeys (*Macaca fascicularis*; S3 and S4), all male, 5.52–8.34 kg (between 4 years 1 month and 9 years 10 months old) at the beginning of behavioural training, participated in this study. Three of the monkeys (S2–S4) were housed socially in troops, in indoor enclosures attached to standard caging; the fourth (S1) was housed individually at the time of testing. Water was available *ad libitum* in the home enclosure; each monkey's daily food ration was delivered in the test box and was supplemented with fruit, and forage mix in the home enclosure. All experimental procedures were conducted under the authority of personal and project licences held by the investigators in compliance with the UK Animals (Scientific Procedures) Act, 1986.

### Apparatus

Behavioural testing took place in an automated apparatus. Each monkey was taken from the home enclosure into the test cubicle in a wheeled transport cage, which was fixed in front of a video display unit with a touch-sensitive screen (380 × 280 mm, 800 × 600 pixel resolution). The monkey could reach through horizontally orientated bars (∼ 45 mm apart) at the front of the cage to reach the screen and the rewards. Stimulus presentation, recording of touches to the screen, and reward delivery were all under computer control. A pellet dispenser delivered 190 mg banana-flavored or sugar pellets (P. J. Noyes, Lancaster, NH, USA) into a food cup located below the touch screen. A metal ‘lunchbox’ (∼ 200 × 100 × 100 mm) was located to the left of the food cup and was filled with the ‘large food reward’ which consisted of a mixture of wet monkey chow, seeds, apple, banana, orange, nuts and dates. Infrared cameras positioned at different locations within the test cubicle permitted observation of the monkey while it was performing the task. The entire apparatus was located in an experimental cubicle that was dark except for the illumination of the video screen.

### Behavioural testing

The object-in-place scene-learning task was adapted from Gaffan (1994). Each trial consisted of an artificially constructed scene that occupied the whole area of the display screen. Two foreground objects, small randomly selected and coloured typographic characters, were each placed in a constant location in the scene. The backgrounds were generated using an algorithm which drew a random number (between two and seven) of randomly located ellipses and ellipse segments of random colour, size and orientation on a randomly coloured initial background, and then drew a single very large randomly selected typographic character, clearly distinct in size from the foreground objects, in a random colour somewhere in the scene. All the colours were assigned with the constraint that the foreground objects should be visible (that is, there was a minimum separation in colour space between the colours of a foreground object and the colour of any element of its local background). In each scene, one of the two foreground objects was the correct one for the monkey to touch and the other was incorrect. Because these scenes were generated by an algorithm based on a random number generator, an infinite number of unique scenes could be generated. For example stimuli, see Browning *et al*. (2005) and Gaffan (1994).

After each monkey learned to touch single foreground objects against a black background, additional scene elements were introduced in shaping programs until the monkey reliably touched the foreground object when presented with a new scene. Problems were then introduced with two foreground objects (one correct and one incorrect, as described above) and the number of scenes given in each session was gradually increased, based on each monkey's performance. In the final version of the task, 20 new scenes were presented in each session; the list of 20 scenes was repeated eight times. A touch to the correct object caused the object to flash for 2 s, then the screen blanked and a reward pellet (190 mg; P. J. Noyes) was delivered. A touch to the incorrect object caused the screen to blank immediately. For the first repetition of the list of scenes only, incorrect responses were followed by a correction trial in which the scene was re-presented with only the correct object present. Touches anywhere else in the scene caused the screen to blank and the trial was repeated. When the monkey completed the final trial of a session the lunchbox opened automatically, and the monkey received the large food reward. If the final trial was incorrect, a correction trial was given so that the monkey only ever received the large food reward following a correct response.

The dependent measure was the number of errors (initial touches of the incorrect foreground object) in each presentation of the list of 20 scenes. One monkey (S2) performed poorly on lists of > 10 scenes during his preoperative training, so he was given 20 repetitions of 10 scenes in each daily session rather than eight repetitions of 20 scenes. Data for this monkey were taken from the first eight repetitions to ensure comparability with the other subjects.

Once performance on the scenes task stabilized each monkey was given a 2-week period of rest, after which he was given 12 daily sessions of testing. Data from the final 10 sessions of this test constituted the preoperative performance test (preop). This test was repeated in the same way beginning at least 2 weeks after the first surgery [postoperative performance test 1 (postop 1)] and again after the second surgery, after which the disconnection was complete (postop 2). The comparison between preop and postop 1 reveals any effects of the unilateral injections into the prefrontal cortex; the comparison between postop 1 and postop 2 reveals the effects of the disconnection of ventrolateral prefrontal cortex from the inferotemporal cortex.

### Surgery

Neurosurgical procedures were performed in a dedicated operating theater under aseptic conditions. Each monkey's first neurosurgical procedure consisted of injections into the left ventrolateral prefrontal cortex (neurotoxin in S1–S3 and vehicle in S4), and each monkey's second procedure was an ablation of the right inferotemporal cortex. Steroids (methylprednisolone, 20 mg/kg) were given (i.m.) the night before surgery, and three doses were given 4–6 h apart (i.v. or i.m.) on the day of surgery, to protect against intraoperative oedema and postoperative inflammation. The monkey was sedated on the morning of surgery with both ketamine (10 mg/kg) and xylazine (0.5 mg/kg) and/or midazolam (0.25 mg/kg), i.m. Once sedated, the monkey was given atropine (0.05 mg/kg) to reduce secretions, antibiotic (amoxicillin, 8.75 mg/kg) for prophylaxis of infection, opioid (buprenorphine 0.01 mg/kg i.v., repeated twice at 4- to 6-h intervals on the day of surgery, i.v. or i.m.) and nonsteroidal anti-inflammatory (meloxicam, 0.2 mg/kg, i.v.) agents for analgesia, and an H2 receptor antagonist (ranitidine, 1 mg/kg, i.v.) to protect against gastric ulceration as a side-effect of the combination of steroid and nonsteroidal anti-inflammatory treatment. The head was shaved and an intravenous cannula put in place for intraoperative delivery of fluids (warmed sterile saline drip, 5 mL/h/kg). The monkey was moved into the operating theater, intubated, placed on isoflurane anaesthesia (1–2.75%, to effect, in 100% oxygen) and then mechanically ventilated. Adjustable heating blankets allowed maintenance of normal body temperature during surgery. Heart rate, oxygen saturation of haemoglobin, mean arterial blood pressure, end tidal CO_2_, body temperature and respiration rate were monitored continuously throughout surgery.

The monkey was placed in a head-holder and the head cleaned with alternating antimicrobial scrub and alcohol and draped to allow a midline or coronal incision. The skin and underlying galea were opened in layers. For the inferotemporal ablations, the right zygoma was removed to improve access to the temporal lobe. The temporal muscles were retracted as necessary to expose the skull surface over the intended lesion site. A bone flap was turned over the desired lesion site (prefrontal or inferotemporal) and the craniotomy was extended with rongeurs as necessary. The dura was cut and reflected over the intended lesion site. When the lesion was complete, the dura was sewn over the lesion site, the bone flap replaced and held with loose sutures, and the skin and galea were closed in layers. The monkey was removed from the head-holder and anaesthesia discontinued. The monkey was extubated when a swallowing reflex was observed, returned to the home cage, and monitored continuously until normal posture was regained (usually within 10 min). Nonsteroidal anti-inflammatory analgesic (meloxicam, 0.2 mg/kg, oral) and antibiotic (8.75 mg/kg, oral) treatment continued following surgery in consultation with veterinary staff, typically for 5 days. Operated monkeys that lived in social groups rejoined those groups as soon as practicable after surgery, usually within 3 days of the operation.

#### Neurotoxic ventrolateral prefrontal lesions

**Fig. 1:**
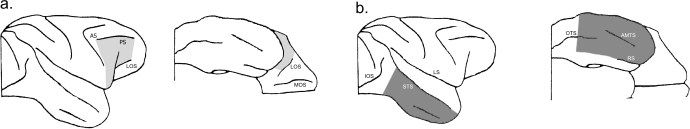
(a) Maximum intended extent of unilateral ventrolateral prefrontal neurotoxic lesions (light grey), showing lateral and ventral views. (b) Intended extent of unilateral inferotemporal ablations (dark grey), also showing lateral and ventral views.

The maximum intended extent of the ventrolateral prefrontal lesions is shown in Fig. 1. It includes the entire ventrolateral surface of the prefrontal cortex anterior to a line drawn between the posterior tip of the principal sulcus and the end of the descending limb of the arcuate sulcus, extending anteriorly to a line drawn between the anterior tip of the principal sulcus and the anterior tip of the lateral orbital sulcus. The dorsal limit of the intended lesion was the ventral lip of the principal sulcus. On the orbital surface the intended lesion in all three cases was to include tissue lateral to the lateral orbital sulcus. Thus, the lesion was intended to remove areas 47/12, 45A and the ventral portion of area 9/46 (Petrides & Pandya, 2002; cf. Rushworth *et al*., 1997). In some cases, as part of the development of this method, we attempted to include tissue between the lateral and medial orbital sulci.

Neurotoxic ventrolateral prefrontal lesions in subjects S1–S3 were produced by multiple 1 µL injections of a mixture of ibotenic acid (10 mg/mL; Biosearch Technologies, Novato, CA, USA) and *N*-methyl-d-aspartic acid (10 mg/mL; Tocris, Bristol, UK) dissolved in sterile 0.1 m phosphate-buffered saline. Subject S4 received injections of phosphate-buffered saline vehicle in precisely the same way. Once the cortical surface was exposed, a stereotaxic manipulator holding a 10-µL Hamilton syringe with a bevelled 26-gauge needle, filled with toxin (S1–S3) or sterile phosphate-buffered saline (S4), was placed over the cortical surface so the tip of the needle was ∼ 2 mm ventral to the ventral lip of the principal sulcus at its posterior tip. The manipulator was angled so that the needle was orientated normally to the surface of the cortex. Injections were made by lowering the needle 4 mm into the cortical surface from the point of the needle on the surface. The injection was made slowly over ∼ 10 s and the needle was left in place for ∼ 15 s before being moved to the next site. Injections continued at 2-mm intervals, moving ventrally from the first injection site, until the exposed cortical surface at that level had been covered. The needle was then advanced 2 mm anteriorly and its tip repositioned 2 mm below the ventral lip of the principal sulcus, and a new series of injections was begun. In this fashion between 21 and 27 injections were placed on the surface of the ventrolateral prefrontal cortex (22 in S1, 24 in S2, 27 in S3 and 21 in S4). In two of the cases further injections were placed anteriorly, by eye with the needle hand-held, when the manipulator was not orientated optimally to place these injections (two in S2 and three in S4). In three of the cases injections were placed through the thickness of the convexity by retracting the brain, measuring the position of the sphenoid bone with the tip of the needle, then advancing the needle through the thickness of the convexity with the coordinate of the sphenoid bone as a guide. Between 5 and 10 injections were placed through the thickness of the inferior frontal convexity in this way (10 in S1, five in S2 and seven in S4) at several anterior–posterior levels. Finally, in three of the cases, a number of injections (four in S2, 13 in S3 and 19 in S4) were made in the orbital surface between the lateral and medial orbital sulci, by retracting the frontal lobe and placing the injections through the hand-held needle. Thus the monkeys received between 27 and 32 injections into the ventrolateral prefrontal cortex (32 in S1, 31 in S2, 27 in S3 and 31 in S4) and between 32 and 50 injections total (32 in S1, 35 in S2, 40 in S3 and 50 in S4) including the orbital injections.

#### Unilateral inferotemporal ablations

The intended extent of the unilateral inferotemporal ablations is shown in Fig. 1, and is identical to such lesions in previous experiments from this laboratory on memory of monkeys following crossed unilateral lesions of frontal and inferotemporal cortex (e.g. Parker & Gaffan, 1998; Browning *et al*., 2005). Cortex of the right temporal lobe was removed extending from the fundus of the superior temporal sulcus to the fundus of the rhinal sulcus. The posterior part of the lesion included both banks of the occipitotemporal sulcus. The posterior limit of the lesion was a line perpendicular to the superior temporal sulcus, 5 mm anterior to the inferior occipital sulcus. The anterior limit of the lesion was bounded by a line drawn from the anterior tip of the superior temporal sulcus around the temporal pole to the tip of the rhinal sulcus. All of the cortex was removed within these limits, including both banks of the anterior and posterior middle temporal sulci. Cortical tissue was removed by subpial aspiration using a small-gauge sucker insulated everywhere except at the tip; electrocautery was applied to remove the pia mater and control bleeding encountered during the ablation.

### Histology

After completion of behavioural training each monkey was sedated with ketamine (10 mg/kg), deeply anaesthetized with intravenous barbiturate and transcardially perfused with 0.9% saline followed by 10% formalin. The brain was cryoprotected in formalin–sucrose and then sectioned coronally on a freezing microtome at 50 µm thickness. A 1-in-10 series of sections through the area of the lesion was mounted on gelatin-coated glass microscope slides and stained with cresyl violet.

The neurotoxic prefrontal lesions were similar in all three cases. The dorsal limit of the lesion was the principal sulcus. Cavitation of cortical tissue was apparent on the surface of the inferior convexity. Cell loss extended into the ventral bank of the principal sulcus in all three cases. The lesion extended more ventrally in cases S2 and S3 than in case S1. Furthermore, several foci of cell loss in the lateral orbital gyrus (area 13) were apparent in case S3, the case that received the most injections into the orbital surface. The anterior–posterior extent of the lesion followed the length of the principal sulcus, as intended. There was some sparing of the most ventral part of the lateral surface in case S1. This sparing of cortex is a result of the gradual development of this improved technique for cortical lesions in monkeys, and a major contribution of the current experiment is the enhancement of our technique for making such injections into prefrontal cortex, especially in less accessible areas such as the orbital surface. All three animals showed, as intended, near-total loss of cells in areas 47/12 and the ventral part of areas 9/46, as well as area 45A and the rostral part of area 45B, although the lesions did not extend into the anterior bank of the descending limb of the arcuate sulcus (Petrides & Pandya, 2002).

**Fig. 2:**
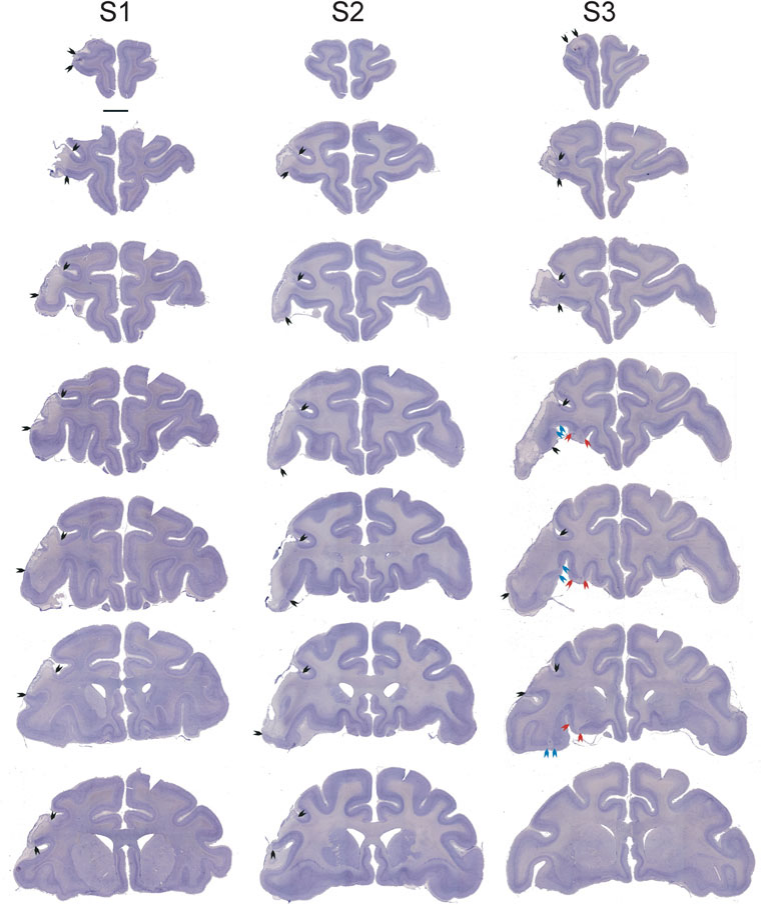
Coronal cresyl violet-stained sections through the frontal lobe in the three cases (S1, S2 and S3) that received injections of neurotoxin into the left ventrolateral frontal cortex. The most anterior section in each case is at the top of the figure and the most posterior at the bottom. Compare the left, lesioned hemisphere of each section with the right, intact hemisphere; arrows indicate the extent of the lesions. Scale bar, 5 mm (applies to all sections).

**Fig. 3:**
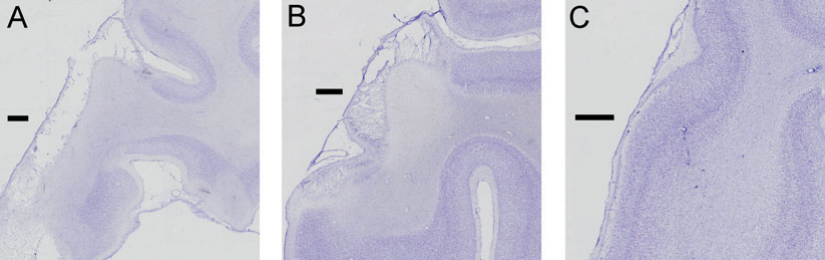
Higher power images at the midpoint of the frontal lobe in three cases. (A) Case S3, illustrating cell loss in the inferior convexity, ventral bank of principal sulcus, and a focus of cell loss in the lateral orbital gyrus. (B) Case S1, illustrating a similar extent of cell loss in the inferior convexity and ventral bank of principal sulcus to case S3. (C) Case S4, which received saline injections into the frontal cortex. No cell loss is evident but a needle track is visible. Scale bars, 1 mm.

**Fig. 4:**
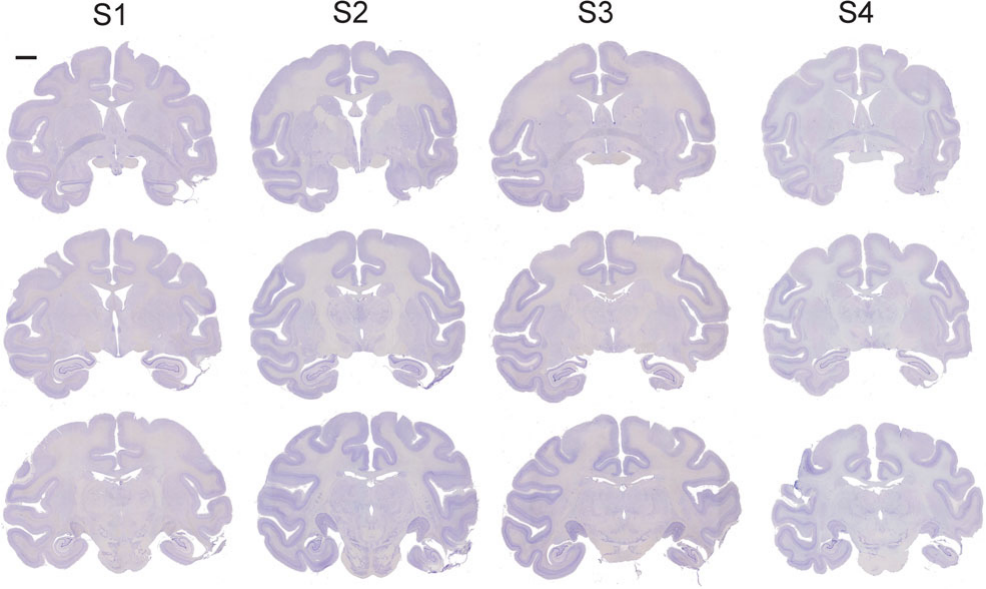
Coronal cresyl violet-stained sections through the temporal lobe in all four cases, illustrating the extent of the inferotemporal ablation. The most anterior section in each case is at the top of the figure and the most posterior at the bottom. Compare the right, lesioned hemisphere of each section with the left, intact hemisphere. Damage to the parietal cortex, apparent in the left hemisphere in the most posterior section for case S4, was sustained in a surgery performed after the data reported in the present paper were collected. Scale bar, 5 mm (applies to all sections).

Figure 2 illustrates a series of coronal sections through the extent of the neurotoxic prefrontal lesion in each case. Higher power photomicrographs taken at the midpoint of the lesion are shown in Fig. 3. This also illustrates the lack of damage to prefrontal cortex produced by vehicle injections in case S4. The inferotemporal lesions were as intended in all four cases and are illustrated in Fig. 4.

## Results

Changes in performance between preop testing and postop 1, and between postop 1 and postop 2, were analysed by repeated-measures anova with testing phase and each trial (repetition) of the list of scenes as factors. Unilateral neurotoxic lesions of ventrolateral prefrontal cortex were without effect on scene learning, based on a comparison of preop and postop 1 phases. This analysis revealed a main effect of trial, as expected (*F*_7,14_ = 36.39, *P* < 0.0005) but no main effect of test phase (*F*_1,2_ = 6.18, *P* = 0.13) or test phase × trial interaction (*F*_7,14_ = 0.51, *P* = 0.81). Analysis of the summary measure of number of errors on trials 2–8 of the lists of scenes revealed identical results (*t*_2_ = 2.19, *P* = 0.16). Thus, there was no overall difference in level of performance or in rate of learning new scene problems after unilateral neurotoxic lesions of ventrolateral prefrontal cortex.

**Fig. 5:**
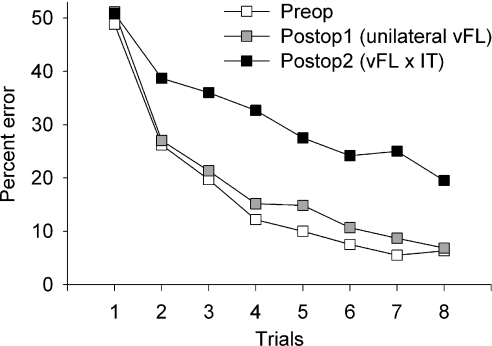
Learning curves for the eight repetitions of lists of new scenes before surgery, after unilateral ventrolateral frontal (vFL) lesions (Postop 1), and after disconnection from inferotemporal cortex (IT) was complete (Postop 2). Data presented are the mean percentage error for each repetition of lists of new scenes for cases S1–S3. There was no effect of unilateral frontal lobe damage on scene learning but a severe impairment is evident after the disconnection of IT from the vFL is completed in the second postoperative phase.

When the ventrolateral prefrontal cortex was disconnected from inferotemporal cortex by placement of an inferotemporal lesion in the opposite hemisphere, however, dramatic deficits in scene learning emerged. Comparison of postop 1 and postop 2 revealed in addition to the main effect of trial (*F*_7,14_ = 30.2, *P* < 0.0005) a main effect of test phase (*F*_1,2_ = 44.77, *P* = 0.022) and a test phase × trial interaction (*F*_7,14_ = 4.00, *P* = 0.013). Analysis of the summary measure of number of errors on trials 2–8 of the lists of scenes revealed identical results (*t*_2_ = 6.37, *P* = 0.024). There was no difference on errors on trial 1 (*t*_2_ = 0.13, *P* = 0.91), indicating that this difference was not due to random variation in the number of errors, made by chance, during the initial encounter with each new list of scenes. Thus, the disconnection of ventrolateral prefrontal cortex (produced by a neurotoxic lesion) and inferotemporal cortex resulted in an overall impairment in performance as well as a slowing of learning rate. These data are presented in Fig. 5.

Of course, it could be the case that the effect of the neurotoxic prefrontal lesion was due to multiple penetrations of the frontal cortex with a needle rather than any specific action of the neurotoxin itself; furthermore, these data, by themselves, do not exclude the possibility that unilateral inferotemporal ablation has a severe effect on scene learning by itself. The latter possibility is argued against by previous data showing that unilateral inferotemporal ablation has no significant effect on scene learning (Browning *et al*., 2005). We investigated the former possibility in case S4, who received saline instead of neurotoxin injections into prefrontal cortex. This monkey performed no differently between his preop testing (15.43% errors on trials 2–8 of learning new scene problems) and his postop 1 test after the saline injections (11.29% errors on trials 2–8). Nor did the placement of an inferotemporal ablation contralateral to the prefrontal saline injections impair his performance (12.43% errors on trials 2–8). Because the results in this single subject were clear and the comparisons in the other three cases could be made by within-subjects analysis, we did not judge it necessary to produce a complete control group of monkeys for this experiment. Thus, ‘sham’ disconnection of the ventrolateral prefrontal cortex from inferotemporal cortex was without effect on scene learning, so we may conclude that the impairments in subjects S1–S3 were a consequence of the crossing of neurotoxic damage to ventrolateral prefrontal cortex with ablation of inferotemporal cortex.

We considered whether poor performance postdisconnection could be attributed to perseveration of initial incorrect responses. On this view, an involvement of the prefrontal cortex in behavioural flexibility could impair scene learning because monkeys continue emitting initial incorrect responses and do not change them in response to feedback. This would predict that performance would be more impaired for scenes in which the initial response was incorrect than for scenes in which the initial response was correct. If trials are subdivided based on whether the initial response to each scene (during its first presentation in the session) is correct (1C) or wrong (1W), there is no differential effect of the disconnection on trials where the first response to the scene is wrong relative to trials on which the first response to the scene is correct. Comparison of responding between postop 1 and postop 2 reveals expected main effects of trial, test phase and 1C/1W, as well as an interaction of 1C/1W with trial, but no interaction of 1C/1W with test phase (*F*_1,2_ = 1.42, *P* = 0.36) or three-way interaction of 1C/1W, test phase and trial (*F*_6,12_ = 1.46, *P* = 0.27). Thus, poor performance following the disconnection cannot be explained by an increased perseverative tendency to continue to respond incorrectly to scenes where the initial response is incorrect.

**Fig. 6:**
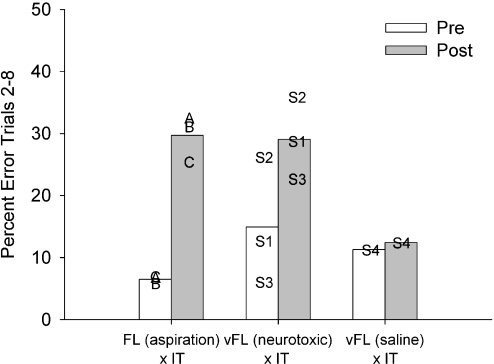
Comparison of neurotoxic ventrolateral frontal lesions, saline injections and full disconnection of frontal lobe from inferotemporal cortex. Data presented are the mean percentage error on trials 2–8 of lists of new scene problems. ‘Pre’ represents performance after unilateral lesions of either the frontal lobe (FL) or temporal lobe (subjects A, B and C in group FL × IT, data from figure 4 of Browning *et al*., 2005) or after unilateral injections into the ventrolateral frontal cortex (subjects S1–S4, groups vFL, present study). ‘Post’ represents performance after frontal–temporal disconnection in all three groups. The impairment in group FL × IT appears to be more severe than that in group vFL (neurotoxic) × IT, although comparison is complicated because of differences in baseline performance. ‘Sham’ disconnection as a result of saline injections into vFL (case S4) was without effect on scene learning.

**Fig. 7:**
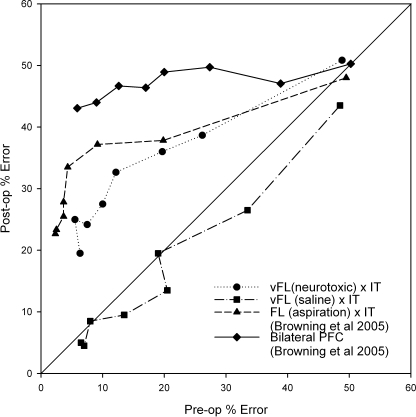
Phase–space plot comparing the current data with those presented in Browning *et al*. (2005). Data are plotted as mean preoperative percentage error against mean percentage error following the second surgeries in the disconnection procedure, or the only surgery in group Bilateral PFC. Each point represents the mean pre- vs. postoperative performance on a given repetition of lists of new scenes. The central diagonal line represents performance that is identical prior to and after surgery. As such, points below this line represent an improvement from pre- to postoperative performance tests, and points above it represent an impairment in performance from pre to post. Group vFL(saline) × IT shows a small improvement, whereas impairments are clear in groups Bilateral PFC, vFL(neurotoxic) × IT, and FL × IT. The impairment in group vFL(neurotoxic) × IT was mildly less severe than that of group FL × IT. FL, frontal lobe; IT, inferotemporal cortex; PFC, prefrontal cortex; vFL, ventrolateral frontal cortex.

It is instructive to compare the current results to those produced by complete disconnection of the frontal lobe from the inferotemporal cortex (rather than just disconnection of the ventrolateral prefrontal cortex). These disconnections were studied by Browning *et al*. (2005) and these and the current data are summarized in Fig. 6. Comparison of Fig. 1 here with fig. 2 of Browning *et al*. (2005) shows that the intended extent of the current unilateral ventrolateral prefrontal lesions falls within the intended extent of the unilateral frontal lesions in that paper. There was no cell loss outside the area ablated by Browning *et al*. (2005), however. Although the final level of performance of the three cases in this study was similar to that of monkeys with full frontal–inferotemporal disconnection, the baseline performance of two of the monkeys in the present study was substantially poorer than that of monkeys in the earlier study. Moreover, case S3, who predisconnection performed similarly to monkeys in the earlier study who went on to receive full frontal–inferotemporal disconnection, was less impaired postoperatively than any of the three monkeys in the earlier study with complete disconnections. Figure 7 is informative when making this comparison. In this phase–space plot, the postoperative and preoperative learning rates are plotted against each other. This means that the severity of the impairments noted in the current study and in Browning *et al*. (2005) can be compared to each other across a range of preoperative performance levels, and hence the comparison can be made without concern over differing levels of preoperative performance between the two studies. Figure 7 supports the previous figures demonstrating the significant impairment in the current experiment but also shows that, across almost all levels of preoperative performance, the impairment observed here was consistently a small amount less severe than that observed following disconnection of the whole of frontal cortex from inferotemporal cortex in Browning *et al*. (2005), and substantially less severe than that observed following bilateral lesions of prefrontal cortex in the same study. The difference between the two disconnection results was, however, small in comparison to the difference between them and the control animal with injections of saline to ventrolateral prefrontal cortex. Thus, although neurotoxic lesions of ventrolateral prefrontal cortex produced a substantial impairment in new scene learning, it appears that they did not fully reproduce the effect of frontal–inferotemporal disconnection on this task. This may imply that either areas outside of ventrolateral prefrontal cortex within the frontal lobe or fibres of passage through the ventrolateral prefrontal cortex participate in scene learning as well.

## Discussion

We have shown that disconnection of a small region of the ventrolateral prefrontal cortex from the inferotemporal cortex, via placement of a neurotoxic lesion in the ventrolateral prefrontal cortex contralateral to ablation of the inferotemporal cortex, produces a substantial impairment in object-in-place scene memory, a macaque model of episodic memory. Previous work from our laboratory has shown that this same task is impaired by a disconnection of the whole of frontal cortex in one hemisphere and the inferotemporal cortex in the other, using aspiration lesions in both cases (Browning *et al*., 2005). The current finding builds on these data to generate two important and novel conclusions. First, the ventrolateral prefrontal cortex is a critical region for episodic-like memory processes in the macaque brain. Second, because neurotoxic cortical damage resulted in substantial impairment in scene learning, one can be more confident that behavioural effects of aspiration lesions within the prefrontal cortex are not a consequence of disconnection of axons moving through the region of the aspiration, at least for lesions within the ventrolateral prefrontal cortex.

There are a number of possible alternative explanations of the current finding, including some that do not refer to episodic-like memory processes. Most notably, earlier studies of the involvement of the ventrolateral prefrontal cortex in memory in macaque monkeys emphasize its role in nonspatial tasks and perseveration. Delayed object alternation, object matching and colour matching were severely impaired following bilateral lesions of the inferior frontal convexity, compared to lesions of the sulcus principalis that produced only transient disruptions in the same tasks (Mishkin & Manning, 1978). However, in another study, lesions of the inferior convexity impaired even simultaneous colour matching; when colour matching was retrained with no delay, subsequent matching performance across delays was unimpaired, implying that the deficit was not one of working memory (Rushworth *et al*., 1997). Instead it may be one of selection of behaviourally relevant stimuli (Rushworth *et al*., 1997, 2005). On this view, impairments in scene learning could result from an impairment in representing relevant features of the scene to guide subsequent performance when confronted with each problem again as the session progresses. We found no differences in inaccurate responses (touches to locations in the scene other than the two target foreground objects) during the first encounter with each scene either after the unilateral prefrontal lesion (preop, mean per 20 scenes 0.7, postop 1, mean 0.30; *t*_2_ = 1.39, *P* = 0.30) or after the disconnection was complete (postop 2, mean 0.8; comparison with postop 1, *t*_2_ = −1.11, *P* = 0.38) indicating that the lesion did not affect the ability of the monkeys to select objects in the scene that were relevant for action (that is, those that were foreground objects, one of which could lead to reward). Similarly, there is no evidence that the impairments in scene learning postdisconnection stem from perseveration to objects chosen during the first encounter.

Other hypotheses have been suggested to account for the effects of frontal–temporal disconnection on memory. For example, the frontal cortex may be required for tasks which require the integration of information across time for their solution. It was suggested that object-in-place scene learning may represent such a task because, in order to process the visual scene, information must be integrated across multiple saccades to take in all of the elements of the scene (Browning *et al*., 2005). Hence, the ventrolateral prefrontal cortex may participate in this function. Our data may also be consistent with a role of inferior prefrontal cortex in ‘controlled semantic retrieval’ (Wagner *et al*., 2001), where memories must be retrieved in the absence of strong stimulus–stimulus or stimulus–response associations. This theory suggests that scene learning is distinct from concurrent object–reward learning, which is unimpaired following frontal–temporal disconnection, because associations between objects and the scenes in which they occur require some top-down influence for retrieval to occur whereas concurrent object–reward associations can be retrieved relatively automatically. These latter two hypotheses are not mutually exclusive, and are both consistent with our interpretation of the current data.

On a more methodological level, this finding suggests that behavioural impairments following aspiration lesions within the prefrontal cortex are not, at least not entirely, due to transection of axons moving through the ablated cortical area. This has been a serious problem in interpreting the effects of aspiration lesions of other structures, for example the amygdala (Baxter & Murray, 2000). However, neurotoxic lesions of the perirhinal cortex have been shown to produce quantitatively identical deficits in recognition memory to those that follow aspiration lesions of the perirhinal cortex (Malkova *et al*., 2001), suggesting that this may not be as critical an issue for interpreting effects of lesions within the prefrontal cortex. Nevertheless, the contribution of white matter damage to impairment following cortical lesions cannot be discounted. Mishkin & Manning (1978) found that monkeys whose lesions invaded the white matter adjacent to the caudate nucleus had much more severe impairments following inferior convexity lesions than did monkeys whose lesions did not include this white matter damage. However, we show that substantial impairment in object-in-place scene learning can be observed when neurotoxic lesions are used that do not damage white matter at all.

Disconnection of ventrolateral prefrontal cortex from inferotemporal cortex appears to produce a milder impairment in object-in-place scene learning than does disconnection of the entire frontal lobe from inferotemporal cortex. It is possible that the incomplete nature of the ventrolateral prefrontal lesions, as discussed in Histology above, may account for this difference. Alternatively, it may imply that other areas of the frontal lobe, outside ventrolateral prefrontal cortex, participate in scene learning; the identification of these areas is a topic of ongoing research. Comparison of the effects of neurotoxic ventrolateral prefrontal lesions with aspiration lesions of ventrolateral prefrontal cortex would allow a test of the hypothesis that deficits consequent to aspiration lesions are due in part to white matter damage, if aspiration lesions of similar anatomical extent to our neurotoxic lesions were found to produce greater behavioural impairment. This hypothesis is also currently under investigation.

In summary, we have shown in this experiment that the ventrolateral prefrontal cortex is essential for rapid learning of new object-in-place scene problems, a form of episodic-like memory in macaque monkeys. By identifying one critical locus for this ability within the macaque frontal cortex, further investigations of the neurochemical substrates of this ability within the frontal lobe now become possible.

## Abbreviations

Postop 1 or 2: postoperative performance test 1 or 2
preop: preoperative performance test
